# Patterns and predictors of e‐cigarette, cigarette and dual use uptake in UK adolescents: evidence from a 24‐month prospective study

**DOI:** 10.1111/add.14723

**Published:** 2019-07-25

**Authors:** Mark Conner, Sarah Grogan, Ruth Simms‐Ellis, Keira Scholtens, Bianca Sykes‐Muskett, Lisa Cowap, Rebecca Lawton, Christopher J. Armitage, David Meads, Laetitia Schmitt, Carole Torgerson, Robert West, Kamran Siddiqi

**Affiliations:** ^1^ School of Psychology University of Leeds Leeds UK; ^2^ Department of Psychology Manchester Metropolitan University Manchester UK; ^3^ Centre for Health Psychology, Science Centre Staffordshire University Stoke‐on‐Trent UK; ^4^ Manchester Centre for Health Psychology, Division of Psychology and Mental Health, Manchester Academic Health Science Centre University of Manchester Manchester UK; ^5^ Institute of Health Sciences University of Leeds Leeds UK; ^6^ School of Education Durham University Durham UK; ^7^ Department of Health Sciences University of York York UK

**Keywords:** Adolescents, cigarette, dual smoking, electronic cigarettes, UK, prospective study

## Abstract

**Background and Aims:**

To assess prevalence and predictors of e‐cigarettes/cigarettes patterns of use in adolescents in England.

**Design:**

Prospective study with 24‐month follow‐up of e‐cigarette/cigarette ever/regular use with data from an intervention evaluation.

**Setting:**

Forty‐five schools in England (Staffordshire and Yorkshire).

**Participants:**

A total of 3210 adolescents who, at baseline, were aged 13–14 years and had never used e‐cigarettes/cigarettes.

**Measurements:**

Based on e‐cigarette/cigarette ever use at follow‐up, six groups were created: (a) never user, (b) e‐cigarette only, (c) cigarette only, (d) dual use—order of use unclear, (e) dual use—e‐cigarettes used first and (f) dual use—cigarettes used first. Baseline measures were: gender, ethnicity, socio‐economic status, impulsivity, family plus friend smoking and smoking‐related beliefs (attitude and perceived behavioural control).

**Findings:**

In groups (a) to (f), there were 71.5, 13.3, 3.3, 5.7, 2.9 and 3.4% adolescents, respectively. Among groups using cigarettes, regular smoking was more prevalent in group (f) (dual use—cigarettes used first) [17.6%, 95% confidence interval (CI) = 10.4, 24.8] than in groups (c), (d) and (e) combined (7.3%, 95% CI = 4.7, 9.9). Among groups using e‐cigarettes, regular use was less prevalent in group (b) (e‐cigarette only) (1.9%, 95% CI = 0.6, 3.2) than in groups (d), (e) and (f) combined (12.2%, 95% CI = 8.9, 15.5). Higher impulsivity plus friends and family smoking were predictive of being in groups (b) to (f) compared with group (a) (never users). Males were more likely to be in group (b) compared to group (a); females were more likely to be in groups (c) to (f) compared to group (a).

**Conclusions:**

Regular use of e‐cigarettes/cigarettes varies across groups defined by ever use of e‐cigarettes/cigarettes. Interventions targeted at tackling impulsivity or adolescents whose friends and family members smoke may represent fruitful avenues for future research.

## Introduction

Electronic cigarettes (e‐cigarettes) represent a means to deliver inhaled aerosol (usually containing nicotine) to the lungs. E‐cigarettes have been recognized as a means to reduce harm in adult smokers 1, 2, 3, 4. In adolescent groups from western countries, the last few years have witnessed increased rates of e‐cigarette use at the same time as rates of cigarette smoking have fallen 5. This is particularly true in the United States 6 and United Kingdom 2, 7, where rates of e‐cigarette use in adolescents are now substantial (13–22%). Nevertheless, the use of e‐cigarettes in adolescent non‐smokers has raised concerns about the impact on progression to starting smoking cigarettes and more regular smoking 8, 9, 10, 11. A limited number of longitudinal studies have examined the predictors of using cigarettes, e‐cigarettes and dual use (both cigarettes and e‐cigarettes) in samples of adolescents who were initially all non‐users 12, 13, 14, 15, 16. These studies 12, 14 identify attitudes and descriptive norms as consistent predictors. However, no study has examined differences between dual users who start with e‐cigarettes versus dual users who start with cigarettes. Such information could add to our understanding of different patterns of smoking/vaping initiation in terms of determinants and consequences and inform interventions.

The present research is novel in examining differences between six groups of 15–16‐year‐old adolescents (all of whom neither used cigarettes nor e‐cigarettes at 13–14 years): never users of cigarettes or e‐cigarettes; e‐cigarette only users; cigarette only users; dual users—order of first use unclear; dual users—e‐cigarettes used first; and dual users—cigarettes used first. Previous reports of the current data focused on examining how e‐cigarette use as a 13–14‐year‐old predicted progression to cigarette smoking at age 14–15 years 10 or 15–16 years 17. The present research tested differences among these six groups. Our aims were to: (1) estimate the numbers in each of the six groups; (2) test differences in the rates of regular cigarette smoking among the four smoking groups (i.e. cigarette only users; dual users—order of first use unclear; dual users—e‐cigarettes used first; dual users—cigarettes used first); (3) test differences in the rates of regular e‐cigarette use among the four e‐cigarette groups (i.e. e‐cigarette only users; dual users—order of first use unclear; dual users—e‐cigarettes used first; dual users—cigarettes used first); and (4) identify the predictors of being in one of the five user groups (i.e. e‐cigarette only users; cigarette only users; dual users—order of first use unclear; dual users—e‐cigarettes used first; dual users—cigarettes used first) compared to the never user group. The research provides insights into the determinants and consequences of different patterns of adolescent use of cigarettes and e‐cigarettes. The former might be useful in generating targeted interventions to reduce smoking initiation in different groups of adolescents, while the latter might be useful in deciding on which groups to target with interventions.

## Methods

### Design

To address the study aims, data from a 4‐year cluster randomized controlled trial of a school‐based smoking initiation intervention 18, 19 based on implementation intentions was used 20. The intervention showed significant effects on reducing smoking initiation (ever smoking a cigarette and any smoking in the last 30 days) but not on regular smoking (smoked a cigarette in the last week) 21. The data reported here are from waves 3 (September–December 2014; referred to as ‘baseline') and 5 (September 2016–January 2017; referred to as ‘follow‐up') of the trial when e‐cigarette use measures were added to the data collection. Only adolescents who self‐reported never using cigarettes or e‐cigarettes at baseline are included in the current report. The effects of intervention condition are controlled for in the analyses (see below). The analyses focus on the six groups (never users of cigarettes or e‐cigarettes; e‐cigarette only users; cigarette only users; dual users—order of first use unclear; dual users—e‐cigarettes used first; and dual users—cigarettes used first) at follow‐up based on ever use of e‐cigarettes/cigarettes.

### Participants and procedures

Data from 3210 adolescents from 45 schools in England (Staffordshire and Yorkshire) who self‐reported never having used a cigarette or an e‐cigarette at age 13–14 years are reported here. Head teachers consented to school participation with parents given the option to withdraw children from the study. Adolescents consented by completing questionnaires matched across time‐points using a personally generated code. The University of Leeds, UK (Faculty of Medicine) ethical review committee approved the study (reference 12–0155).

### Measures

#### Outcomes

Cigarette use was assessed using a standardized measure 22 at both time‐points; adolescents ticked one of: ‘I have never smoked; I have only tried smoking once; I used to smoke sometimes, but I never smoke cigarettes now; I sometimes smoke cigarettes now, but I don't smoke as many as one a week; I usually smoke between one and six cigarettes a week; and I usually smoke more than six cigarettes a week'. Marking the first response versus other responses was coded to indicate never smoking versus ever smoking cigarettes, while marking the last two responses was coded to indicate regular use of cigarettes.

E‐cigarettes/vaporizers were described as ‘a tube that sometimes looks like a normal cigarette and has a glowing tip. They all puff a vapour that looks like smoke but unlike normal cigarettes, they don't burn tobacco'. Use of e‐cigarettes was tapped by a single item at both time‐points [‘Which *one* of the following is closest to describing your experience of e‐cigarettes or vapourizers', I have never used them; I have tried them once or twice; I use them sometimes (more than once a month but less than once a week); I use them often (more than once a week)']. Marking the first response versus other responses was coded to indicate never versus ever using e‐cigarettes, while marking the last responses was coded to indicate regular use of e‐cigarettes.

Among those who reported ever using both cigarettes and e‐cigarettes at follow‐up we assessed whether cigarettes or e‐cigarettes were used first or the order of first use was not recalled. These different measures of smoking at follow‐up were used to create six groups: never users (reported never using cigarettes or e‐cigarettes); e‐cigarette only users (reported using e‐cigarettes at least once but never using cigarettes); cigarette only users (reported using cigarettes at least once but never using e‐cigarettes); dual user—order of first use unclear (reported using e‐cigarettes at least once and cigarettes at least once plus being unsure of the order of use); dual user—e‐cigarettes used first (reported using e‐cigarettes at least once and cigarettes at least once plus using e‐cigarettes first); and dual user—cigarettes used first (reported using e‐cigarettes at least once and cigarettes at least once plus using cigarettes first).

#### Predictors

Based on previous research, nine covariates were measured at baseline and used as predictors of being in the never user group versus the other five user groups. Gender, ethnicity (self‐reported classification dichotomized into non‐white versus white), individual‐level socio‐economic status (four‐item Family Affluence Scale 23) were measured. The personality dimension of impulsivity was also measured (four‐item measure 24; i.e. tendency to act on a whim, displaying behaviour characterized by little or no forethought, reflection or consideration of consequences). Family smoking was assessed using the question: ‘Who smokes in your family now? Tick all the people who smoke at the moment', followed by a list of family members (scored 0–10). Friends' smoking was assessed using the question: ‘How many of your friends smoke?'—none of them; only a few; half and half; most but not all; and all of them (scored 1–5). Two components of health cognitions about smoking 19 were assessed, each being scored on a five‐point scale (high scores indicated negative views of smoking): (i) attitude was tapped by seven questions (‘For me, smoking would be… good–bad; beneficial–harmful; pleasant–unpleasant; enjoyable–unenjoyable; wise–foolish; fun–not fun; healthy–unhealthy'; Cronbach's alpha = 0.87); (ii) perceived behavioural control was tapped by three questions (‘I am confident I could resist smoking', strongly disagree to strongly agree; ‘For me to not smoke would be…', difficult–easy; ‘How much control do you feel you have over not smoking?', no control–complete control; Cronbach's alpha = 0.69). Additional health cognitions that might overlap conceptually with those reported here were not included in the analyses (i.e. intentions, norms, self‐efficacy), although their inclusion did not substantively change the findings. Intervention condition was also included in the analyses.

### Data analysis

In order to assess the study aims we: (1) report the frequencies and percentages in each of the six groups; (2) use χ^2^ analyses to compare rates of regular use of cigarettes across the four groups using cigarettes; (3) use χ^2^ analyses to compare rates of regular use of e‐cigarettes across the four groups using e‐cigarettes; and (4) use multinomial logistic regression to identify the predictors of being in one of the five user groups compared the never used group. We report overall model fit (*R* ^2^, χ^2^) along with the odds ratio and 95% confidence intervals for each predictor. Although not a focus of the present analyses, we report the effect of intervention condition in the multinomial logistic regression and assessed the significance of any interactions between condition and each of the predictors. The analyses did not control for the clustering of data by schools (school was the unit of intervention) due to the limited numbers in each of the user groups in some schools. Sensitivity analyses assess whether similar findings were obtained when imputing missing values on the predictors or when replacing the multinomial logistic regression with five individual logistic regressions (i.e. predicting being in the never user group compared to each of the other five user groups). There were no missing values on user group. We used multiple imputation to estimate missing values on predictors [range of missing values ranged between 0 (0%) for gender and 79 (2.5%) for ethnicity; a total of 103 additional cases included in the analyses after multiple imputation]. Five imputed data sets were created and multinomial logistic regressions averaged across the data sets. All analyses were conducted in SPSS version 24. Full data are available from the first author.

## Results

### Description of groups

In relation to our first aim (estimating the size of different user groups at 15–16 years), we observed that the majority of the sample remained as never users. Adolescents who initiated e‐cigarette use only comprised the second largest group, followed by the dual user (12.0%) and cigarettes only groups. The dual user group split into dual user—order of first use unclear, dual user—e‐cigarettes used first and dual user—cigarettes used first (Table 1).

**Table 1:** Descriptive data for the six user groups (*n* = 3210).

	Cigarette use			E‐cigarette use			
	Never	> Never & < regular	Regular^a^	Never	> Never & < regular	Regular^b^	
Group	n%	n%	n%	n%	n%	n%	Total (% of total)
Never user	2294 (100.0%)	0 (0.0%)	0 (0.0%)	2294 (100.0%)	0 (0.0%)	0 (0.0%)	2294 (71.5)
E‐cigarettes only	425 (100.0%)	0 (0.0%)	0 (0.0%)	0 (0.0%)	417 (98.1%)	8 (1.9%)	425 (13.3)
Cigarettes only	0 (0.0%)	101 (94.4%)	6 (5.6%)	107 (100.0%)	0 (0.0%)	0 (0.0%)	107 (3.3)
Dual user—order of first use unclear	0 (0.0%)	168 (91.8%)	15 (8.2%)	0 (0.0%)	165 (90.2%)	18 (9.8%)	183 (5.7)
Dual user—e‐cigarettes used first	0 (0.0%)	86 (92.5%)	7 (7.5%)	0 (0.0%)	80 (86.0%)	13 (14.0%)	93 (2.9)
Dual user—cigarettes used first	0 (0.0%)	89 (82.4%)	19 (17.6%)	0 (0.0%)	92 (85.2%)	16 (14.8%)	108 (3.4)

Percentages are within‐group (cigarette use or e‐cigarette use) and sum to 100% within a row.

a χ^2^ test of differences in regular cigarette smoking: all four cigarette smoking groups (χ^2^_(3)_ = 10.82, *P* = 0.013); cigarette only, dual user—order of first use unclear and dual user—e‐cigarettes used first groups (χ^2^_(2)_ = 0.68, *P* = 0.713); dual user—cigarettes used first group versus cigarettes only group (χ^2^_(1)_ = 7.51, *P* = 0.006); dual user—cigarettes used first group versus cigarettes versus dual user—order of first use unclear group (χ^2^_(1)_ = 5.81, *P* = 0.016); dual user—cigarettes used first group versus dual user—e‐cigarettes used first (χ^2^_(1)_ = 4.50, *P* = 0.034). ^b^χ^2^ test of differences in regular e‐cigarette use: all four e‐cigarette‐using groups (χ^2^_(3)_ = 37.40, *P* < 0.001); dual user—order of first use unclear, dual user—e‐cigarettes used first, dual user—cigarettes used first groups (χ^2^_(2)_ = 1.91, *P* = 0.384); dual user—order of first use unclear versus e‐cigarettes only group (χ^2^_(1)_ = 19.77, *P* < 0.001); dual user—cigarettes used first group versus cigarettes versus e‐cigarettes only group (χ^2^_(1)_ = 33.50, *P* < 0.001); dual user—cigarettes used first group versus e‐cigarettes only group (χ^2^_(1)_ = 28.70, *P* < 0.001).

In relation to our second and third aims (rates of regular use of cigarettes and e‐cigarettes across user groups), Table 1 reports the relevant findings. Regular use of cigarettes across the cigarette smoking groups ranged from 5.6 to 17.6% and significantly differed across the four groups. Further examination indicated that there were no differences in regular cigarette use between the cigarette only, dual user—order of first use unclear and dual user—e‐cigarettes used first groups. However, the dual user—cigarettes used first group had significantly higher rates of regular cigarette use (17.6%, 95% confidence interval (CI) = 10.4, 24.8) than the other three groups combined (7.3%, 95% CI = 4.7, 9.9) (Table 1).

Rates of regular use of e‐cigarettes across the e‐cigarette user groups ranged from 1.9 to 14.8% and also significantly differed across the four groups (Table 1). Further examination indicated that there were no differences in regular e‐cigarette use across the three dual user groups (i.e. dual—order of first use unclear; dual user—e‐cigarettes used first; dual user—cigarettes used first). However, the e‐cigarettes only group (1.9%, 95% CI = 0.6, 3.2) had significantly lower rates of regular e‐cigarette use than each of the three other groups combined (12.2%, 95% CI = 8.9, 15.5) (Table 1).

### Predictors of group membership

In relation to our fourth aim, Table 2 reports the findings from the multinomial logistic regression for each of the eight predictors plus condition. The model fit was reasonable. Gender, impulsivity and friends smoking emerged as consistent predictors of membership of each of the five groups that used cigarettes and/or e‐cigarettes compared to the never user group. Higher levels of impulsivity and friends smoking were each associated with being more likely to be in one of the five user groups. Males were significantly more likely to use e‐cigarettes only, while females were significantly more likely to be in each one of the four cigarette smoking groups (*P*s < 0.05). In addition, higher levels of family smoking were associated with being significantly more likely to be in four of the five user groups (not dual user—e‐cigarettes first) compared to the never user group. Less consistent patterns were observed for attitudes, perceived behavioural control and intervention condition. More negative attitudes towards smoking (cigarettes only plus dual user—order of first use unclear), less perceived behavioural control over not smoking (dual user—cigarettes first) and being in the intervention condition (dual user—order of first use unclear) were found to reduce the likelihood of belonging to some user groups compared to the never user group (Table 2).

**Table 2:** Multinomial logistic regression results predicting different smoking groups compared to never user (cigarettes or e‐cigarettes) group (*n* = 3107).

	Never user (cigarettes or e‐cigarettes) versus:				
	E‐cigarettes only	Cigarettes only	Dual user—	Dual user—	Dual user—
			order unclear	e‐cigarettes first	cigarettes first
Predictors	OR (95% CI)	OR (95% CI)	OR (95% CI)	OR (95% CI)	OR (95% CI)
Female	1.000	1.000	1.000	1.000	1.000
Male	1.640 (1.320, 2.039)	0.644 (0.424, 0.978)	0.696 (0.501, 0.968)	0.503 (0.314, 0.804)	0.592 (0.383, 0.915)
White	1.000	1.000	1.000	1.000	1.000
Not white	0.727 (0.522, 1.013)	1.040 (0.586, 1.846)	1.163 (0.748, 1.809)	0.531 (0.240, 1.175)	1.162 (0.655, 2.061)
Family affluence	0.978 (0.912, 1.049)	1.059 (0.924, 1.214)	1.002 (0.903, 1.112)	1.028 (0.889, 1.190)	0.916 (0.808, 1.040)
Impulsivity	1.263 (1.183, 1.349)	1.452 (1.286, 1.638)	1.539 (1.396, 1.697)	1.623 (1.422, 1.853)	1.777 (1.558, 2.026)
Friend smokers	1.483 (1.208, 1.821)	1.546 (1.081, 2.211)	2.207 (1.708, 2.851)	1.528 (1.037, 2.252)	1.824 (1.293, 2.573)
Family smokers	1.169 (1.071, 1.277)	1.187 (1.012, 1.391)	1.320 (1.173, 1.485)	1.181 (0.996, 1.400)	1.284 (1.103, 1.496)
Attitudes	0.806 (0.543, 1.196)	0.572 (0.327, 0.999)	0.513 (0.339, 0.776)	0.567 (0.313, 1.025)	0.707 (0.369, 1.354)
Perceived behavioural control	0.921 (0.726, 1.170)	0.922 (0.601, 1.416)	0.922 (0.663, 1.283)	0.735 (0.498, 1.085)	0.697 (0.489, 0.993)
Control group	1.000	1.000	1.000	1.000	1.000
Intervention group	1.114 (0.893, 1.388)	0.760 (0.508, 1.136)	0.717 (0.521, 0.988)	1.021 (0.661, 1.578)	1.229 (0.807, 1.873)

Model fit: *R* ^2^ = 0.140 (Cox & Snell), 0.162 (Nagelkerke). Model χ^2^_(45)_ = 463.607, *P* < 0.001. OR = odds ratio; CI = confidence interval.

Ethnicity and family affluence did not emerge as significant predictors for entering any one of the five user groups compared to the never user group. In addition, there were no significant interactions between condition and any of the predictors. Although the six groups differed considerably in size, the magnitude of the odds ratios for each predictor of being in one of the five user groups compared to the never user group was generally similar (Table 1). The clear exception was in relation to gender, where males were significantly more likely to be in the e‐cigarettes only group, but females were more likely to be in each of the other four user groups, compared to the never user group.

Sensitivity analyses showed that the findings were substantively unchanged by imputing missing values on predictors or by running five separate logistic regressions.

## Discussion

In relation to our four aims, the present study found a number of differences between our six groups (never users; cigarette only users; e‐cigarette only users; dual users—order of first use unclear; dual users—e‐cigarettes used first; dual users—cigarettes used first). Relating to our first aim, never users and e‐cigarette only users were found to be the largest groups among adolescents. It is notable that only using e‐cigarettes was the largest of the five groups who used cigarettes and/or e‐cigarettes confirming other studies showing relatively high rates of e‐cigarette use in UK adolescents 2, 7. In relation to our second aim, regular use of cigarettes across the four cigarette smoking groups varied considerably (5.6–17.6%); it was significantly higher in the dual user—cigarettes used first group compared to each of the other groups. It is notable that regular smoking is higher in this group than the group who only smoke cigarettes, i.e. use of e‐cigarettes alongside cigarettes did not reduce regular smoking. It may be that the dual user—cigarettes used first group contains a larger proportion of dedicated smokers whose experience they seek to enhance with e‐cigarette use. In relation to our third aim, regular use of e‐cigarette use among the four e‐cigarette groups also varied considerably (1.9–14.8%) and was significantly higher in the e‐cigarettes only group compared to each of the other groups. This is perhaps less surprising, given that those in the three dual user groups could have satisfied any craving for nicotine from either regularly using cigarettes or e‐cigarettes while those who only use e‐cigarettes would have only satisfied any craving by regularly using e‐cigarettes. It would be useful for further research to confirm these patterns of use in larger samples before too much reliance is placed on these findings. Nevertheless, the findings would initially appear to support the view that using e‐cigarettes first is associated with lower rates of progression to regular cigarette smoking by age 15–16 years.

Related to our fourth aim, the multinomial logistic regression analyses (Table 2) indicated that, compared to the never user group, the predictors of being in each of the five user groups were generally similar. Friends smoking, family smoking, impulsivity and gender each emerged as consistent independent predictors across groups. In particular, friends and family smoking were consistent predictors of being in each of the user groups, i.e. higher levels of friends and family smoking were associated with being more likely to use e‐cigarettes only, use cigarettes only or use both e‐cigarettes and cigarettes (all three dual user groups) compared to never users. Family smoking was not a statistically significant predictor of being in the dual user—e‐cigarettes used first group, although the odds ratio was of comparable magnitude to that found for other user groups and is probably attributable to this being the smallest user group. Unfortunately, rates of friends and family use of e‐cigarettes were not assessed and we were therefore unable to assess such effects on patterns of adolescent smoking initiation (see 13, 16).

Higher levels of impulsivity were also associated with being more likely to be in each of the user groups compared to the never user group. The effects for impulsivity were somewhat smaller for e‐cigarette only use. Males were statistically significantly more likely to be in the e‐cigarette only group compared to the never user group, while females were statistically significantly more likely to be in each of the other smoking groups compared to the never user group. The fact that e‐cigarette use appears to be appealing more to males than females deserves further attention, as it is not clear what is the basis of these differences.

Ethnicity, family affluence, perceived behavioural control and intervention condition were generally not predictors of being in one of the user groups compared to the never user group. Finally, positive attitudes towards not smoking were associated with not being a member of each of the user groups (this effect attained statistical significance only for the cigarettes only and the dual user—order unclear user groups) compared to the never user group.

In general, the data (Table 2) did not suggest strong differences between user groups in terms of the longitudinal predictors of being in each user group compared to the never user group. In particular, we failed to observe differences in the predictors of being in the dual user—cigarettes used first group compared to being in the never user group versus being in the dual user—e‐cigarettes used first group compared to being in the never user group. However, this may be attributable to the relatively small numbers in these dual user groups. In addition, we did not assess various predictors of e‐cigarette use employed in other studies, such as perception of the harmfulness of e‐cigarettes, attitudes towards e‐cigarettes, and others' use of e‐cigarettes 12, 13, 14, 15, 16.

The most novel aspect of the present research was the examination of differences between dual user—cigarettes used first and dual user—e‐cigarettes used first groups. The predictors of these different patterns of dual smoking were very similar across the set of variables examined. Other research has reported that low levels of friends smoking may be associated with a greater likelihood of transitioning between using e‐cigarettes and cigarettes 9, 10. More detailed examination of a broader range of predictors of different patterns of dual use (e‐cigarettes first versus cigarettes first versus use of both at same time) in larger samples would be useful.

Our study has a number of strengths, including a large demographically diverse sample, measurement of e‐cigarette and cigarette use over 24 months, and exploration of a large set of covariates including socio‐demographic variables as well as measures of health cognitions about smoking. There are, however, also a number of weaknesses. First, our study focused on self‐reported e‐cigarette and cigarette use. Secondly, we failed to distinguish types of e‐cigarette use (e‐cigarettes vary in a number of ways including the delivery method and whether they contain nicotine). Thirdly, our research did not include measures of perceptions of e‐cigarettes shown to predict e‐cigarette and dual use 12, 13, 14, 15, 16. Fourthly, we did not ask about e‐cigarette use among family and friends to assess their influence on e‐cigarette/cigarette use compared to non‐users. Fifthly, we did not ask about age at which cigarette and/or e‐cigarette use began thus precluding any consideration of duration. Sixthly, it is possible that the many dual users could not accurately recall whether they used cigarettes or e‐cigarettes first, which increases the uncertainty of the findings. It could be that this ‘unsure' group is distinct and first used cigarettes and e‐cigarettes at a similar point in time or that they more accurately belong in one of the dual user—cigarettes used first or dual user—e‐cigarettes used first groups. Finally, our research had a limited geographical (two English counties) and age (baseline: 13–14 years) distribution and did not control for the clustering of the data by school (schools‐level randomization to intervention and control conditions). Nevertheless, there are no strong reasons to suspect that any of these factors would have substantially altered the findings reported. Future studies might address some of these issues and explore effects in different aged adolescents and over varying time‐periods.

In summary, this is the first study, to our knowledge, to report longitudinal relationships between different patterns of e‐cigarette and/or cigarette use among UK adolescents. A key recommendation would be to focus on preventing the initiation of cigarette use because this, irrespective of subsequent e‐cigarette use, may lead to increased regular cigarette use.

## Declaration of interests

All authors report receiving grants from the National Prevention Research Initiative during the study. The authors have no conflicts of interest.
